# Placing joint hypermobility in context: traits, disorders and syndromes

**DOI:** 10.1093/bmb/ldad013

**Published:** 2023-06-22

**Authors:** Silvia Morlino, Marco Castori

**Affiliations:** Division of Medical Genetics, Fondazione IRCCS-Casa Sollievo della Sofferenza, San Giovanni Rotondo, Italy; Division of Medical Genetics, Fondazione IRCCS-Casa Sollievo della Sofferenza, San Giovanni Rotondo, Italy

**Keywords:** Beighton score, Ehlers-Danlos syndrome, hypermobility spectrum disorders, international classification, joint hypermobility

## Abstract

**Background:**

Joint hypermobility (JHM) is a common physical trait. It may occur alone or in combination with musculoskeletal (MSK) pain, outside or within more complex phenotypes. Hypermobility spectrum disorders (HSD) are diagnosed in individuals with JHM and related MSK pain, when an alternative diagnosis cannot be identified. Conversely, the Ehlers-Danlos syndrome (EDS) encompasses a group of rare hereditary connective tissue disorders featuring JHM along with other pleiotropic manifestations. The 2017 EDS Classification identifies 13 different subtypes. Hypermobile EDS (HEDS) is the only EDS variant still lacking a confirmatory test.

**Sources of data:**

Literature was reviewed searching for the most relevant papers related to key arguments. Particular attention was focused on papers published after the 2017 Classification.

**Areas of agreement:**

Definition, epidemiology, assessment tools and patterns of JHM are presented. The morbid nature of the 2017 EDS Classification and of the ‘spectrum’ is also illustrated.

**Areas of controversy:**

We discuss current limitations and disagreements concerning the ‘spectrum’, HSD and HEDS.

**Growing points:**

In the clinical context, elucidation of the pathophysiology of pain related to JHM should develop in parallel with the analysis of pleiotropic manifestations of syndromes with JHM.

**Areas timely for developing research:**

Future challenges concerning classification, nosology, diagnosis and management of JHM, EDS and related disorders are discussed.

## Introduction

Ehlers-Danlos syndrome (EDS) encompasses a clinically variable and genetically heterogeneous group of rare soft connective tissue disorders chiefly characterized by joint hypermobility (JHM), easy bruising, abnormal wound healing, and altered skin texture presenting with soft, velvety, doughy and/or hyperextensible skin.^1^ The International Classification of EDS and related disorders published in 2017 identifies thirteen clinical subtypes, which are distinguished according to the differential expression of the main phenotypical hallmarks, presence of additional distinctive features and/or inheritance pattern. The combined variability of these elements influence the overall severity of the disease and the pattern of patients’ needs. Accurate diagnosis is essential for an evidence-based approach to EDS and related disorders.

Among the various pleiotropic manifestations which characterize the EDS ‘core phenotype’, JHM has stimulated growing attention in the last two decades. Although the cutaneous and vascular aspects of the disease dominated the medical literature in the last century due to their immediate and sometimes dramatic impact in terms of mortality, JHM and its relationship with chronic symptoms were highlighted only recently in the context of EDS, thanks to the seminal work by dr. Voermans and collaborators on pain and fatigue.^2^^,^^3^ In these papers, the authors emphasized a high rate of chronic musculoskeletal (MSK) and systemic symptoms in individuals with EDS, which have a significant impact on quality of life (QoL). Since then, a multitude of papers corroborated the generally poor QoL of people with specific EDS subtypes^4^; a fact that cannot be easily linked to the cutaneous and vascular manifestations, but rather seems to associate with JHM.

Indeed, recognition of the potential detrimental effect of JHM on the MSK system goes back to the middle of the past century, when dr. Kirk and collaborators pointed out an association between JHM and MSK pain,^5^ originally called ‘hypermobility syndrome’. The term earned increasing popularity at the beginning of the current century, thanks to the publication of the revised diagnostic criteria for the ‘joint hypermobility syndrome’ (JHS).^6^ The clinical similarities between JHS, EDS and, in particular, the hypermobility-type of EDS (HT-EDS) prompted a group of experts to state that JHS and HT-EDS should be considered one and the same condition until molecular discoveries allow them to be separated on the basis of more robust data.^7^ However, this opinion was not shared by all researchers.^8^

At that time, the revised criteria for JHS (‘Brighton criteria’)^6^ and the Villefranche criteria for HT-EDS^9^ were strikingly overlapping. The inefficacy of these two sets of diagnostic criteria in identifying discrete phenotypes was formally demonstrated in a family study in which both aggregated in the same pedigrees.^10^ Moreover, the use of the Beighton score (BS)^11^ as the tool for assessing JHM in both sets of criteria did not help the medical community to separate the concepts of JHM, JHS and HT-EDS in their minds. In fact, a single ‘letter’ (‘r’ vs ‘e’) distinguishes the Brighton criteria for diagnosing a clinically relevant condition (i.e. JHS) from the BS for measuring a (semi-)quantitative physical trait (i.e. JHM).

While the phenotypic boundaries of the other subtypes of EDS are determined by the identification of causative variants in the associated genes, this is not possible for JHS and HT-EDS which remain without a known molecular basis. In the last decade, the assumption that JHS and HT-EDS cannot be distinguished from the one another, and the incorporation of the latter into the EDS nosology generated an ‘explosive increase’ of EDS cases in many countries. While EDS is recognized as a rare disease, the association between MSK pain and JHM is quite common, particularly in specific sub-populations. This generated a paradox, and nurtured the false impression of a commonality of medical needs among individuals with syndromic presentations of JHM and those with non-syndromic JHM combined with MSK pain.

## JHM: definition and epidemiology

JHM describes the ability that a joint has to move beyond its normal limits. The adjective ‘normal’ refers to the mean range of motion (ROM) observed in the general population for any given joint, along physiological axes. Such a variability strictly relates to the dominant, evolution-driven attributes of the human MSK system.^12^ In particular, major anatomical and physiological contributors to joint mobility include health of the articular surfaces, underlying bone morphology, muscle tone, and integrity of the soft tissues around joints, including ligaments, tendons and capsules. Sex, age and ethnicity may affect significantly the contribution of each of these components to the eventual ROM in any given individual by the intersection of a mixture of congenital/developmental and acquired/degenerative processes. A series of modifiable factors may also influence the ROM and includes, for example, nutritional status, weight/body composition, working activities, training, past traumas and surgery. In addition, an effective assessment of joint ROM often relies on the active collaboration of the evaluated individual. For this reason, the developmental, mental and general health status of the patient can influence the validity of physician’s measurements. JHM is defined by ROM that extends beyond the mean physiological values. Although JHM is an attribute of a single joint or group of joint, its pattern distribution within the MSK system is an attribute of the individual (see below). In related systemic diagnoses, the pattern of distribution of JHM is often a key diagnostic feature, rather than the identification of JHM ‘per se’. In the medical literature, synonyms for JHM include joint laxity and joint hyperlaxity.^13^

**Fig. 1:**
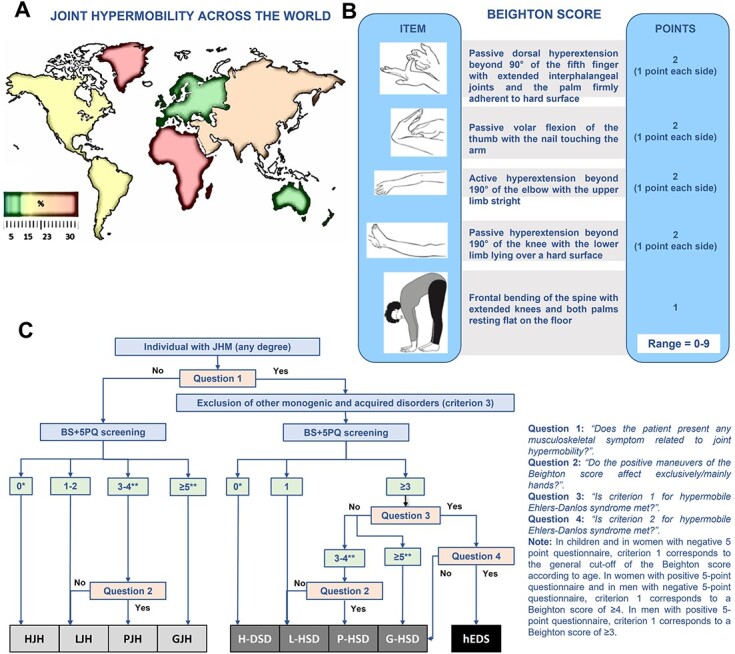
(**A**) Prevalence of joint hypermobility worldwide. An overview of the prevalence of joint hypermobility in various populations and countries. (**B**) BS. When possible, the ROM should be measured with an orthopedic goniometer. Adapted from: https://www.ehlers-danlos.com/. The cut-off for generalized joint hypermobility is 5 and 6 for adults and children, respectively (ref. no. 18). (**C**) Decision tree for individuals with joint hypermobility within the ‘spectrum’. ^*^Historical joint hypermobility/HSD needs a positive 5PQ. ^*^^*^In children, the BS cut-off for generalized joint hypermobility is increased by 1 (= 6). 5PQ, five-point questionnaire. BS, Beighton score. GJH, generalized joint hypermobility. G-HSD, generalized hypermobility spectrum disorder. hEDS, hypermobile Ehlers-Danlos syndrome. HJH, historical joint hypermobility. H-HSD, historical hypermobility spectrum disorder. LJH, localized joint hypermobility. L-HSD, localized hypermobility spectrum disorder. PJH, peripheral joint hypermobility. P-HSD, peripheral hypermobility spectrum disorder.

**JHM is common in the general population and is often a benign trait without significant health-related manifestations**. Observation indicates an overt excess of JHM in females (females = 6-57% vs males = 2-35%),^14^ children and adolescents. The rate of JHM is also affected by ethnicity. It is more common in Inuit, African and Asian people and less common in Europe and Australasia. Intermediate rates are recorded in North, Central and South America (Fig 1A).^14–16^ Therefore, the ‘a priori’ significance that should be attributed to JHM in a clinical setting is strongly influenced by the background non-modifiable factors of the assessed individual.

### JHM: assessment

Assessment of ROM and, consequently, JHM now rarely forms part of the routine physical examination in most clinical settings. Recognizing JHM needs a clinical suspicion and appropriate tools and methods. JHM is a clinically relevant sign in the context of MSK pain or syndromic suspicion. Therefore, an unexplained recurrent/chronic pain which cannot be attributed to any other rheumatologic, neurologic and neuromuscular disorder should elicit a search for specific patterns of JHM (see below). At the same time, looking for specific patterns of JHM should be a component of any systematic physical examination in clinics specialized in the diagnosis of genetic disorders. In fact, JHM and, more specifically, generalized JHM (see below) is a feature of many genetic disorders, such as hereditary connective tissue disorders.^17^

Accurate assessment of JHM needs the use of measurement tools, including the orthopedic goniometer and the flexible tape, and a reference set of values according to the most common non-modifiable factors, including sex, age and ethnicity. Normal reference ranges adjusted for every variable are not available, but there are many specialized publications presenting reference sets and the Centers for Disease Control and Prevention offer some online resources (ps://www.cdc.gov/ncbddd/jointrom/index.html). Assessment of multiple joints is necessary to explore the relationship between JHM and the reported pain, and to plan an adequate treatment program. Distinguishing the distribution pattern of JHM within the whole body of the examined subject (see below ‘joint hypermobility: patterns’) is a prerequisite for using diagnostic decision tools.

Currently, the most widely accepted tool for standardizing assessment of JHM distribution is the BS which was originally conceived as an epidemiological resource for evaluating JHM in young Africans.^11^ The BS includes the evaluation of nine joints or group of joints. In the presence of clinically appreciable JHM, 1 point is attributed to each manoeuvre, while 0 points are assigned in case of normal ROM. A BS of at least 5 or 6 is accepted as the cut-off for generalized JHM in adults or children, respectively (Fig. 1B).^18^ Concerns remain about the reliability of the BS in recognizing generalized JHM in real world medicine. In fact, the cited cut-offs do not consider the distribution of positive manoeuvres (e.g. right vs left body halves, upper vs lower body halves), acquired limitations (e.g. immobilized or amputated limbs), variability among ethnicities and between sexes, or any co-existing JHM in other joints. The BS is current preferred because it is easy-to-use and does not require particular training, but ideally should be employed as part of a more extensive assessment of ROM. There are other, more complete and detailed tools, including the upper limb hypermobility assessment tool for adults^19^ and the lower limb assessment score for adults and children^20^^,^^21^ suitable for therapists, which evaluate a greater number of joints and, therefore, could be considered also for diagnostic purposes in selected scenarios. Their application in the diagnostic setting has not been validated yet.

JHM can be a synonym for congenital contortionism or ‘double-jointedness’ and can be lost over the years as the result of growth, senescence, changes in life habits, surgery, traumas, etc. In these circumstances, it is termed historical (generalized) JHM and cannot be assessed objectively. In 2003, Hakim and Grahame developed a short questionnaire (the five-point questionnaire; 5PQ) to use for evaluating a past history of generalized JHM.^22^ A positive response to at least two of the following questions is considered indicative for historical (generalized) JHM: *(a) can you now (or could you ever) place your hands flat on the floor without bending your knees?; (b) can you now (or could you ever) bend your thumb to touch your forearm?; (c) as a child did you amuse your friends by contorting your body into strange shapes or could you do the splits?; (d) as a child or teenager did your shoulder or kneecap dislocate on more than one occasion?; (e) do you consider yourself double-jointed?*. Currently, the 5PQ is used as a support tool for establishing criterion 1 of hypermobile EDS (hEDS) in the 2017 International Classification of EDS and related disorders (see below).^23^

### JHM: patterns

Pattern analysis of JHM identifies four categories including generalized JHM, peripheral JHM, localized JHM and historical JHM.^13^

Generalized JHM is the most extensive form of JHM and affects multiple joints (or groups of joints) of the appendicular and axial skeleton. It is reliably recognized by a positive BS and is typically congenital. Generalized JHM is the pattern of JHM most commonly associate with single gene disorders.

Peripheral JHM is a form of JHM that affects the extremities (i.e. hand and foot joints) only. It is often bilateral and is quite common in children. In peripheral JHM, BS is negative. Peripheral JHM is frequently a benign trait with a relatively low significance in terms of a syndromic suspicion. However, some exceptions exist and include, for example, vascular and myopathic EDS, and genomic disorders such as the 47,XXY male. Therefore, the diagnostic relevance of peripheral JHM is strongly influenced by the clinical context in which it is identified.

Localized JHM affects a single or a few (≤2) joints (or group of joints). The BS is negative by definition. Compared with generalized and peripheral JHM, localized JHM is more commonly acquired due to, for example, traumas and surgery.

Historical JHM is a form of JHM that is confined into the past history of the patient. Currently, it can be explored mainly with the 5PQ.^22^ Nevertheless, its clinical relevance outside the context of generalized, peripheral or localized JHM is questioned.

### Joint hypermobility and Ehlers-Danlos syndromes

In humans, JHM can be considered in three broad contexts: (i) as a common **physical trait** influenced by or contributing to sexual dimorphism,^12^ (ii) representing a **predisposing factor** to transient and chronic MSK dysfunctions, symptoms and traumatic events (see below), and (iii) as part of a **syndromic** diagnosis. A contemporary approach to JHM must consider each context in turn. Intuitively, the well-recognized higher prevalence of joint laxity in toddlers, children and women cannot be interpreted as being a consequence of a Mendelian trait and/or of hormonal diversity only.^12^ As recently proposed, the phenomenon of JHM in health and disease can be more easily conceptualized by applying the integration model.^12^

Although Mendelian inheritance explains genetic syndromes with JHM as rare events driven by single highly-penetrant variants in pleiotropic genes, the integration model recognizes less specific phenotypes as emerging from the more complex, fluctuating interactions of a multitude of effectors (e.g. multilocus inheritance, sex, ethnicity, in-utero development, post-natal development, lifestyle, etc.). Mendelian inheritance and the integration model should be viewed as collaborating forces.^12^

The number of genetic syndromes featuring (generalized/peripheral) JHM as a penetrant feature is large and includes hereditary soft connective tissue disorders, selected skeletal dysplasias, several hereditary muscle disorders, some multiple congenital anomalies/intellectual disability syndromes, and specific chromosomal and genomic disorders.^13^ Among them, the EDSs are the most prominent. This is likely due to a higher penetrance of JHM in conditions caused by pathogenic variants in genes encoding proteins directly involved in the homeostasis of the extracellular matrix (ECM), which is the core constituent of ligaments, tendons and joint capsules. Given the modular nature of the human body and its development, constitutional alterations of the ECM tend to present with generalized/peripheral JHM rather than with other more restricted patterns. At the same time, pain is currently conceived as mechanistically associated with JHM, which, in turn, predisposes to MSK complaints by still incompletely known biomechanical, biochemical and neurological processes.^24^ Therefore, the mechanism by which JHM combines with pain is not necessarily related to pleiotropy.

The 2017 International Classification of EDS and related disorders identifies 13 distinct clinical subtypes due to deleterious variants in 19 different genes.^23^ All of them are rare diseases (conditions that affect less than five individuals in 10 000 in Europe, or fewer than 200 000 people in USA) and the most prevalent subtypes include classical,^25^ vascular^26^ and hEDS.^27^ All EDS subtypes except for hEDS have one or more known causative genes. The hEDS of the 2017 International Classification substitutes the HT-EDS of the Villefranche nosology and is identified by more stringent clinical diagnostic criteria (an easy-to-use check-list for the diagnosis of hEDS is freely downloadable at: https://www.ehlers-danlos.com/heds-diagnostic-checklist/). As hEDS is still without a robust diagnostic biomarker, molecular testing (see below) is useful for ruling out differential diagnoses when there is diagnostic uncertainty (criterion 3).

After the publication of the International Classification, a fourteenth type of EDS resembling classic EDS but inherited in an autosomal recessive pattern was described associated with biallelic variants in the *AEBP1* gene.^28^ Two years later, renewed attention focused on the so-called osteogenesis imperfecta/EDS overlap due to specific variants in *COL1A1* and *COL1A2*. Although excluded from the International Classification, a recent case series and clinical reports indicates a possible fifteenth type of EDS, temporarily termed *COL1*-related overlap disorder,^29–31^ to be included in future revisions of the International Classification.

Table 1 shows recognized clinical subtypes of EDS and associated major criteria. Each clinical subtype also has a set of minor criteria, not reported in the table, whose presence supports the clinical suspicion. At present, the minimum set of major and/or minor criteria indicating molecular testing is not well defined for all EDS clinical subtypes. However, for vascular EDS, molecular testing is indicated in the presence of at least one major criterion or ‘several’ minor criteria particularly in individuals younger than 40 years,^32^ whereas, in classical EDS, the diagnosis is established by the presence of ‘minimal clinical diagnostic criteria’ and the identification of a causative variant in an associated gene.^33^

**Table 1:** Known clinical subtypes of the Ehlers-Danlos syndrome

**Type**	**Gene(s)**	**Protein(s)**	**Inheritance pattern**	**Estimated prevalence**	**Major diagnostic criteria**
*Subtypes included in the 2017 International Classification of Ehlers-Danlos syndromes and related disorders*					
Classical	*COL5A1, COL5A2, COL1A1* [p.(Arg312Cys)]	α1(V) procollagen, α2(V) procollagen, α1(I) procollagen	AD	~1/20.000	Skin hyperextensibility with atrophic scarring Joint hypermobility (generalized)
Classical-like (type 1?)	*TNXB*	Tenascin-X	AR	Unknown (rare or ultrarare)	Skin hyperextensibility with velvety skin in the absence of atrophic scarring Joint hypermobility (generalized) with or without recurrent dislocations Easy bruising
Cardiac-valvular	*COL1A2* (null alleles)	α2(I) procollagen	AR	Unknown (rare or ultrarare)	Severe progressive cardiac-valvular problems Skin involvement (skin hyperextensibility, atrophic scars, thin skin, easy bruising) Joint hypermobility (variable)
Vascular	*COL3A1, COL1A1* [p.(Arg312Cys), p.(Arg574Cys), p.(Arg1093Cys)]	α1(III) procollagen, α1(I) procollagen	AD	1/50.000-200.000	Family history of vascular EDS with identified variant in *COL3A1* Arterial rupture at a young age Spontaneous colonic rupture in the absence of an underlying disease Uterine rupture during the third trimester in the absence of C-section or perineum tears Carotid-cavernous sinus fistula in the absence of trauma
Hypermobile	Unknown	Unknown	AD (?)	<1/5.000	Joint hypermobility (generalized) Systemic involvement Positive family history Musculoskeletal involvement Exclusion of other diagnoses (see text)
Arthrochalasia	*COL1A1* (skipping of exon 6), *COL1A2* (skipping of exon 6)	α1(I) procollagen, α2(I) procollagen	AD	Unknown (rare or ultrarare)	Congenital bilateral hip dislocation Severe joint hypermobility (generalized) with multiple dislocations/subluxations Skin hyperextensibility
Dermatosparaxis	*ADAMTS2*	Procollagen I N-procollagen	AR	Unknown (rare or ultrarare)	Extreme, congenital skin fragility Characteristic craniofacial features Postnatal growth retardation Redundant/lax skin with excessive skin folds Increased palmar wrinkling Easy bruising (severe) with increased risk of subcutaneous hematomas and hemorrhages Umbilical hernia Postnatal growth retardation Short limbs/hands/feet Perinatal complications due to tissue fragility
Kyphoscoliotic	*PLOD1, FKBP14*	Lysyl-hydroxylase 1, FKBP22	AR	Unknown (rare or ultrarare)	Congenital muscle hypotonia Congenital/early-onset kyphoscoliosis Joint hypermobility (generalized) with dislocations/subluxations
Brittle cornea syndrome	*PRDM5, ZNF469*	PR-domain containing protein 5, zinc finger protein 469	AR	Unknown (rare or ultrarare)	Thin cornea with or without rupture Easy-onset, progressive keratoconus Early-onset, progressive keratoglobus Blue sclerae
Spondylodysplastic	*B3GALT6, B4GALT7, SLC39A13*	Galactosyltransferases I and II, ZIP13	AR	Unknown (rare or ultrarare)	Short stature Muscle hypotonia Bowing of limbs
Musculocontractural	*CHST14, DSE*	Dermatan-4-O-sulfotransferase 1, dermatan sulphate epimerase 1	AR	Unknown (rare or ultrarare)	Congenital contractures (mainly, adducted thumbs, talipes equinovarus) Characteristic facial features Skin involvement (skin hyperextensibility, easy bruising, atrophic scarring, skin fragility, increased palmar wrinkling)
Myopathic	*COL12A1*	α1(XII) procollagen	AD	Unknown (rare or ultrarare)	Congenital muscle hypotonia and/or atrophic which improves with age Joint contractures (proximal) Joint hypermobility (distal)
Periodontal	*C1R, C1S*	Complement subcomponents 1r and 1 s		Unknown (rare or ultrarare)	Early-onset, severe and intractable periodontitis Lack of attached gingiva Pretibial plaques Family history of a first degree relative who meets the criteria
*Candidate Novel Clinical Subtypes*					
Classical-like (type 2?)	*AEBP1*	AE-binding protein 1	AD	Unknown (rare or ultrarare)	Not yet defined
*COL1*-related overlap disorder	*COL1A1, COL1A2*	α1(I) procollagen, α2(II) procollagen	AD, AR (rare)	Unknown (rare or ultrarare)	Blue sclerae Flatfeet with valgus deformity of the hindfoot Joint hypermobility (generalized) Significantly soft and doughy, and/or hyperextensible skin

AD, autosomal dominant. AR, autosomal recessive.

Assessing people for possible EDS is a time-consuming process and requires training. ROM evaluation needs familiarity with the use of an orthopedic goniometer and BS computation. Identifying skin texture and scarring anomalies with a minimal risk of over- and under-reporting is difficult because the boundaries between clinical sign and normal variability are not well defined. Finally, in many scenarios, ancillary investigations are required to complete the assessment. A full array of practical considerations for the assessment of people with EDS is reported by Malfait and co-authors.^1^

## Genetic testing

Given the increased use of next-generation sequencing technologies in most high-income countries and the high rate of positive findings in highly selected patients’ cohorts,^34^ molecular testing is currently mandatory for the diagnosis of all EDS subtypes except for hEDS. Therefore, the diagnosis of monogenic forms of EDS can be established only by the identification of a causative genotype. In most circumstances, molecular screening targets single nucleotide variants (SNV), large indels and copy number variations (CNV).

Sometimes, individuals with clear-cut phenotypes receive negative or inconclusive results at comprehensive molecular testing by standard diagnostics. Among the most clinical challenging medical genetics reports are those consisting of variants of unknown significance (VUS) or incomplete genotypes (e.g. a single heterozygous pathogenic or likely pathogenic variant in an autosomal recessive gene) in candidate gene(s). Within the EDS nosology, negative and incomplete results are more likely due to suboptimal sensibility of the applied technique(s) or too narrow molecular differential diagnosis (or, perhaps, misdiagnoses), rather than locus heterogeneity extending to still unknown genes. These cases can receive a ‘provisional clinical diagnosis’^23^ by tertiary diagnostic centres and should be periodically reviewed for (i) further molecular testing which should consider genocopies (e.g. Loeys-Dietz syndromes in cases with an original suspicion of vascular EDS), or (ii) innovative diagnostic pipelines improving the detection of alternative molecular mechanisms, such as intragenic rearrangements, deep intronic variants, regulatory variants and low-level mosaicism, or supporting the pathogenicity of previously identified VUS.

Useful investigations to support molecular testing include immunoassay for serum dosage of tenascin X in *TNXB*-related classic-like EDS, dermis ultrastructural analysis in classical, dermatosparaxis and arthrochalasis subtypes, gel electrophoresis analysis from skin fibroblasts for collagen I- and III-related phenotypes, and quantification of deoxypyridinoline and pyridinoline crosslinks in urine with high-performance liquid chromatography in kyphoscoliotic EDS.^1^ Minigene reporter assay, RNA study and transcriptome analysis help in characterizing variants predicted to alter the splicing machinery.

Molecular testing is regularly requested by tertiary centres with genetic expertise, such as Clinical Genetics, or by services dedicated to JHM and hereditary connective tissue disorders. While automated pipelines are routinely introduced into the workflow of diagnostic molecular genetics laboratories, stringent attribution of the American College of Medical Genetics criteria, variant selection for reporting and identification of appropriate actions needed according to available clinical information still require a ‘human interface’, whose effectiveness is strongly influenced by the degree of expertise in the specific thematic area of the laboratory personnel.

### JHM and MSK pain

The association of JHM and MSK pain does not currently constitute a ‘genetic syndrome’. The term ‘spectrum’ is now used to define the variable association of JHM and MSK pain outside the ‘constraints’ of the Mendelian forms of EDS. In this context, pain is considered a detrimental effect of JHM within the MSK system (pathogenesis) and, therefore, not necessarily the direct consequence of a mutated gene (pleiotropy).

Epidemiologically, pain appears to be the most relevant clinical issue related to JHM. A PubMed search with the terms {pain AND ‘joint hypermobility’} retrieved 422 results (February 15, 2022). Early medical literature identified a link between MSK pain and JHM in the propensity to macro- and micro-traumas to which the innate or acquired ligamentous laxity predisposes a hypermobile joint.^5^ In the last decade, an increasing number of publications are supporting a much more complicated picture with a variety of documented or still speculative mechanisms (potentially) explaining the various features of pain in JHM-related phenotypes. A recent paper reviewed mechanisms, models and challenges of pain in EDS.^24^ In order to address such a hard task, the authors extensively reviewed past publications and also included papers on JHS and hypermobility spectrum disorders (HSD), which are currently recognized as phenotypes in continuity but nosologically separated from EDS (see below). In this review, the authors pointed out that all known types of pain (i.e. nociceptive, neuropathic and nociplastic) contribute to QoL in people with EDS (mainly hEDS, see below) and HSD.^24^ JHM-related MSK pain, particularly if associated with generalized JHM, has a protean and still incompletely understood natural history.^35^ Pain often starts in joints with more marked ligamentous laxity, weight-bearing joints (e.g. hips, knees and ankles) and joints more predisposed to multidirectional forces (e.g. shoulders). In the early phases, pain is mainly nociceptive and related to repetitive (subclinical) microtraumas or traumatic events such as dislocations and soft-tissue injuries. Both micro- and macro-traumas are facilitated by lack of proprioception and reduced muscle strength and endurance, all findings frequently encountered in children and adults with JHM.^24^ Neuropathic pain may progressively superimpose on nociceptive pain. Possible mechanisms include entrapment, compression and peripheral neuropathies, and small fibre neuropathy.^36–39^ Nevertheless, none of these factors, either in isolation or in combination, seem to explain the full spectrum of neuropathic features reported in hEDS and HSD. In some individuals, pain subsequently become less and less localized with a tendency to assume a widespread and chronic character. In this phase, people present various symptoms related to pain sensitivity (e.g. allodynia and hyperalgesia) with a clear overlap with fibromyalgia.^40^ Other types of non-MSK pain, such as headache, abdominal pain and dyspareunia, as well as JHM-related co-morbidities, such as depression, anxiety, and gastrointestinal functional disorders, complicate the more advanced/dysfunctional phenotypes.^13^^,^^24^

### Assessing individuals with spectrum disorders

HSD are currently at the intersection of non-syndromic/asymptomatic JHM (ns/aJHM), which defines benign forms of JHM without clinical relevance (at least, at the time of evaluation), and hEDS, as defined according to the criteria of the 2017 International Classification of EDS and related disorders.^13^ HSD are a clinically variable and etiologically unexplained group of phenotypes featuring JHM presenting in its various patterns (generalized, localized, peripheral, and, perhaps, historical) and MSK pain, in the absence of any recognizable genetic syndrome. In people with HSD, molecular testing is either not indicated or negative by definition and the hEDS diagnostic criteria are not met. Although some of these criteria may be present, they are insufficient for a clinical diagnosis of hEDS. Figure 1(C**)** presents the decisional flow-chart for guiding physicians within the ‘spectrum’ including ns/aJHM, HSD and hEDS. Currently, HSD is the commonest diagnosis among individuals with clinically relevant JHM.

**Fig. 2:**
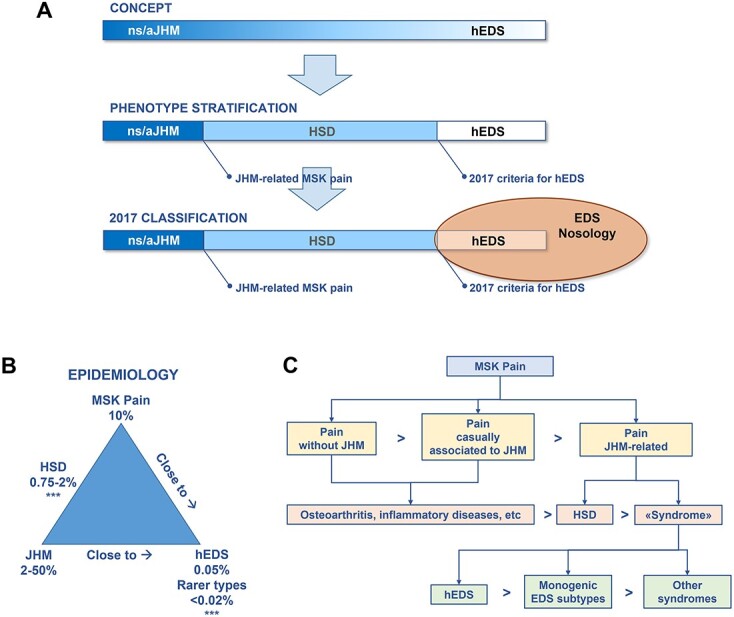
(**A**) Conceptualization of ‘the Spectrum’ and progression into the 2017 Classification of EDS and Related Disorders. ‘The Spectrum’ is a theoretical concept incorporating all phenotypes of JHM related to its association with MSK features and outside the pleiotropic nature of monogenic syndromes. On one end, there are the various forms of non-syndromic, asymptomatic JHM (ns/aJHM). On the other end, they progressively intermingle with symptomatic and more complex, though still molecularly undefined, phenotypes, whose extreme is represented by the hEDS. In the clinical context, the presence of JHM-related MSK symptoms, mainly pain, separates ns/aJHM from the HSD. At the same time, the 2017 criteria for hEDS distinguish the latter from HSD. hEDS is the only EDS type that remains without known molecular basis. Therefore, its diagnosis is established exclusively by the application of clinical criteria. Conversely, the diagnosis of all other EDS types needs confimation by positive molecular testing or, in selected cases only, alternative investigations. (**B**) Epidemiological overview of key terms in the field of JHM and EDS. In the blue triangle, MSK pain, JHM and EDS are identified as separate entities which may coexist in specific clinical scenarios. The combination of MSK pain and JHM more commonly occurs in the context of HSD. This circumstance should be distinguished from MSK pain which independently occurs in combination with JHM (see ‘C’), as well as from EDS and, in particular, hEDS which commonly features both MSK pain and JHM. The percentages indicate trait/condition frequencies in the general population. ^*^^*^^*^Value presumed in the absence of any robust epidemiological study according to the current view in the field of JHM and EDS. (**C**) phenotypical relationships between MSK pain and JHM. MSK pain may present in combination with various degrees/forms of JHM. Nevertheless, observing MSK pain and JHM in the same individual does not always imply a causal relationship. Therefore, JHM-related MSK pain should be distinguished from the chance co-occurrence of MSK pain and JHM. Among the clinical forms of JHM-related MSK pain, HSD is currently recognized as the commonest one. More rarely, JHM-related MSK pain is observed in the context of genetic syndromes, among which EDS and, in particular, hEDS are the most frequent diagnoses.

The ‘spectrum’ is a term grouping together ns/aJHM, HSD and hEDS as a continuous spectrum of phenotypes in which JHM dominates the physical exam (Fig. 2A) with variability between unrelated individuals, members of the same family, and within the same individual across her/his lifespan. Transition between ns/aJHM to and from HSD (ns/aJHM ↔ HSD) is recognized thanks to the superimposition of different factors triggering or attenuating MSK pain during the life of a hypermobile subject.^41^ At the same time, periodic monitoring of subjects with HSD is indicated, especially if the diagnosis is established at a young age, because some diagnostic criteria for hEDS are influenced by age and therefore a reassessment of the diagnosis may be necessary in the adulthood (HSD → hEDS). Given the potentially transitory nature of JHM-related MSK manifestations and the still unknown mechanisms regulating their evolution during post-natal development, the diagnosis of hEDS should be considered with prudence in children, whereas HSD is a useful label for symptom interpretation and treatment. Currently, the only transition that is not accepted is that from hEDS to HSD or ns/aJHM.^41^ That because hEDS is recognized as a rare constitutional disorder without chance of disappearing, similar to all other EDS subtypes.

During the assessment of people with MSK pain and JHM, the frequency of these traits in the general population should be considered (Fig. 2B). In fact, both chronic pain and JHM are equally common. Therefore, the simple observation of pain and JHM in the same individual does not always indicate a **causal relationship**. A **chance concurrence** should be always considered and appropriately excluded by ruling out other mechanisms of MSK pain, such as rheumatic diseases. In other words, the diagnosis of JHM-related MSK pain is by ‘exclusion’, because, currently, there is no confirmatory test. On the other hand, since JHM itself may result in MSK dysfunction regardless of the underlying ‘cause’, sometimes the complex ‘pain-JHM’ (i.e. JHM-related MSK pain) occurs in the context of syndromes and disorders other than HSD and EDS (Fig. 2C). Therefore, the axes ‘JHM → pain’ and ‘JHM → diagnosis’ should be approached separately and collated only after a systematic examination of all possible combinations.

## Discussion

This paper has focused on some salient concepts, and areas of agreement and controversy around JHM and EDS. It has been conceived to stimulate physicians in refreshing and, perhaps, reorganizing their knowledge in this field, which pertains to many common clinical scenarios. While EDS remains a group of rare and ultrarare disorders with a very small chance of being encountered in non-specialized settings, this is not the case for JHM. The link between JHM and clinically relevant issues, such as MSK pain, are increasingly recognized in the medical literature. Physicians are required to manage the issue with competence in order to avoid both misdiagnoses and underestimation of associated symptoms. The fields of JHM and EDS are full of areas of future development which represent challenges for a new generation of physicians and clinical researchers.

### Classificatory challenges

The definition of JHM is easy to understand. Nevertheless, the reliability of available tools and procedures used for measuring JHM is relatively low, and there are many circumstances in which practitioners can be disoriented. JHM is a fluid phenomenon with a wide range of factors influencing its extent. The BS does not take into account some common variables such as ethnicity, sex, age, training and traumatic events leading to joint immobilization and limb ampulation. In addition, a multitude of joints are not included into the BS computation and this may lead to unexpected under- and over-recognition of generalized JHM. For this reason, physiotherapists have developed additional tools more effective for functional assessment and treatment purposes (see ‘joint hypermobility: assessment’).^42^ Similar upgrades are expected for diagnostic purposes in order to improve and speed up the assessment of individuals with suspected HSD or EDS.

Some causal relationship between JHM and MSK pain is currently accepted in people with pain but without other diagnoses. Nevertheless, mechanisms underlying pain triggering and amplification are not fully understood especially in people with widespread pain or pain spatially unrelated to hypermobile joints. In these subjects, the contribution of nociplastic pain is assumed but the processes underlying such a transition remain unexplained, as does the relationship to other chronic MSK pain conditions, such as fibromyalgia.^24^ Understanding the existence of specific pathophysiological mechanisms and/or some convergence with other rheumatic diseases is crucial for improving treatment strategies and QoL of people with symptomatic JHM.

The 2017 International Classification of EDS and related disorders updated the list of genotypes and phenotypes forming the nosology of EDS, and proposed a novel framework for classifying individuals with JHM when the observed phenotype is not in keeping with the EDS nosology.^13^^,^^23^ The reason for the introduction of HSD was twofold. First, the existence of two set of diagnostic criteria for JHS (Brighton criteria) and HT-EDS (Villefranche nosology) identifying strongly overlapping conditions generated confusion among physicians and patients. Secondly, since the delineation of the new criteria for hEDS, it was clear that a large number of individuals with ‘symptomatic’ JHM would remain without a diagnosis or, perhaps, would receive the wrong diagnosis of hEDS. The concept of HSD recognizes the existence of a broad group of individuals presenting occasional, recurrent or chronic MSK pain pathophysiologically related to JHM but in the absence of a clinical or molecular diagnosis of EDS. In the past medical literature, this was not sufficiently clear and led to the false impression that EDS is a common disorder and that pain is a primary manifestation of the (presumed) underlying genetic defect. Both assumptions are currently considered incorrect.

To date, HSD has more often been used as a descriptor than a formal diagnosis.^41^ Since the publication of the International Classification, The Ehlers-Danlos Society (https://www.ehlers-danlos.com/) has been working hard to improve our understanding of phenotypic boundaries and natural history of HSD and its nosologic relationship with hEDS. At present, the ‘diagnosis’ of HSD is established by observing MSK pain in combination with JHM and after the exclusion of any other more clearly established diagnoses. Some phenotypic and historical elements may support the ‘stringency’ of a diagnosis of HSD especially in the presence of pain patterns not ‘typical’ for JHM.^41^ The provision of a standardized tool (such as a set of diagnostic criteria) for confirming the diagnosis in clear-cut presentations and guiding the physician in more complex scenarios is expected for the future. Some preliminary but robust data indicate that the separation between HSD and hEDS is not a matter of ‘severity’.^43^^,^^44^ Other factors, such as the severity and complexity of extra-MSK functional symptoms (i.e. psychological distress, psychiatric co-morbidities, developmental coordination disorders, functional gastrointestinal disorders/symptoms, orthostatic intolerance), likely determine patients’ needs. In addition, the natural history of HSD and, more clearly, of JHM-related MSK pain is only partly known. Although many individuals report the typical progression from occasional nociceptive joint pain to chronic widespread MSK pain,^24^^,^^35^ there are people who experience localized and occasional pain for years and decades, and others who respond with complete and persistent pain relief to treatment (see below). The lack of a direct relationship between closely related diagnoses and the observed severity and required interventions is a not uncommon in medical practice. Maintaining a distinction between HSD and EDS is prudent and recognizes the need for defining key areas of uncertainty for future research.

### Diagnostic challenges

Currently, HSD is the most likely diagnosis for individuals with (various degrees of) JHM and otherwise unexplained MSK pain. The ‘four questions’ reported in Figure 1(C) help the clinician to rapidly screen their patients within the ‘spectrum’. Similarly, the diagnosis of hEDS is also clinical but according to a set of positive and negative criteria. Molecular testing is useful in the doubtful cases or in the presence of one or more features suggestive of a specific Mendelian disorder.

Next-generation sequencing has revolutionized research and diagnostics of genetic disorders in the last decade. Nevertheless, short-reads technology and bioinformatics pipelines applied to routine diagnostics do not still cover the entire spectrum of possible molecular mechanisms altering known disease-genes. In addition, current knowledge is limited concerning locus and clinical heterogeneity in evolving syndromes such as EDS. In fact, there are still cases with full-blown clinical characteristics with negative or incomplete molecular results despite the application of gold-standard strategies and individuals with complex phenotypes with pending diagnoses due to variants in unknown genes. The identification of novel disease-genes, more detailed genotype–phenotype correlations and innovative diagnostic workflows will help in improving accuracy and efficacy of laboratory diagnostics in EDS and related disorders.

### Management challenges

**Fig. 3:**
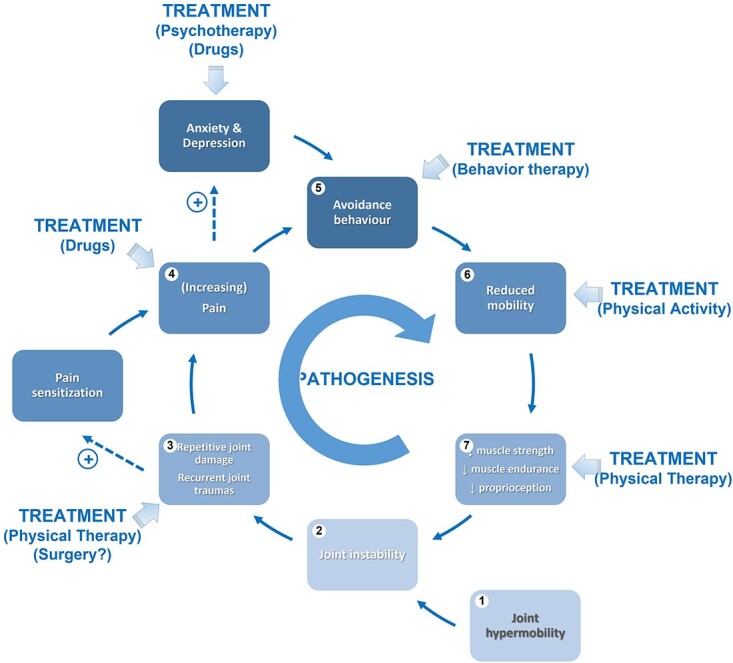
Simplified representation of the pathogenetic processes leading from JHM to a repetitive cycle causing chronic MSK pain and worsening disability in JHM-related MSK pain.

Treating JHM-related MSK pain can be difficult because people are often referred to the ‘expert’ after years of evolving symptoms. Current understanding of the mechanisms which underlie JHM-related MSK pain identifies a progression from soft tissue/joint traumas facilitated by JHM, to pain chronicity, to fluctuating disability (Fig. 3). Treatment follows an integrated, biopsychosocial approach^42^ that considers pain as the summation of a multitude of intermingled mechanisms. Available strategies, such as physiotherapy, pain-killer drugs and cognitive-behavioral therapy, are not always effective in the medium and long terms, and accessibility to tailored programs is not guaranteed in all healthcare systems. The availability of patient pathways emerging from the coordinated work of transnational initiatives, such as the European Reference Networks for rare and complex diseases and international foundations like the Ehlers-Danlos Society, facilitates standardization of treatment accessibility and delivery within the various healthcare systems. Similar needs are found for the syndromic patient who requests periodic follow-up for the early detection and treatment of late-onset complications related to the pleiotropic manifestations of the identified genetic mutation.

Cardiovascular and hollow organ involvement and, in particular, the risk of life-threatening events represent the most severe, though relatively rare manifestation of specific EDS subtypes. Spontaneous arterial ruptures are a major feature of vascular EDS but may occur at lower rates in other EDS clinical subtypes.^45^ Preliminary genotype–phenotype correlations suggest a variable severity of cardiovascular involvement in vascular EDS according to the mutation type.^33^ Much less information is available for the other EDS subtypes with a documented or presumed increased risk of vascular events.^46^ Given the unpredictability of arterial and hollow organ ruptures in EDS, and the complexities generated by the tissue fragility in the emergency room, risk prediction according to patients’ stratification and availability of risk reduction procedures are aims for the future. The identification of innovative drugs for treating pain and improving tissue fragility should become an emerging field of pre-clinical and clinical research in EDS and related disorders.

## Conclusions

JHM is a phenomenon with intricate ramifications throughout different aspects of human variability. Assessment of a minimum set of joints, at least including the BS and other large joints should be routine in many clinical scenarios, particularly those encountered in Rheumatology, Physical Medicine and Rehabilitation, pediatric subspecialties and Clinical Genetics. Intrinsic limitations of joint mobility measurements and factors influencing their reliability must be recognized to effectively discriminate between neutral and clinically relevant JHM. Coexistence of JHM and MSK symptoms is common in the general population, is rarely related to an underlying monogenic connective tissue disorder, and needs prompt recognition for appropriate management independently from the background diagnosis. HSD is currently the ‘default’ diagnosis for people with JHM and related MSK pain once other acquired/monogenic disorders have been excluded. Differential diagnosis prior to the diagnosis of HSD always requires rheumatologic workup, whereas molecular testing for excluding monogenic disorders is indicated in selected cases only. Molecular testing for monogenic disorders featuring JHM should be performed by medical genetics laboratories with expertise in the field. In addition, as JHM is common in the general population, the exclusion of a chance concurrence should be always considered especially those with unusual symptoms not directly related to an underlying dysfunctional MSK system. The current nosology of EDS and related disorders still displays some areas of uncertainty especially related to the ‘spectrum’. The phenotypic continuity between HSD and hEDS nurtures the hypothesis that HSD and EDS may be one and the same disorder. Although the etiology and pathogenesis of HSD (and, perhaps, hEDS) seems to be much more heterogeneous than rarer EDS subtypes. Ongoing and future studies will help in better classifying and stratifying patients with HSD for maximizing health systems resources and with the ultimate hope of substantially improving the patient QoL.

**Silvia Morlino (MD, PhD)** is a consultant in Medical Genetics in the Division of Medical Genetics at Fondazione IRCCS-Casa Sollievo della Sofferenza (Italy). Her clinical and research interests include hereditary connective tissue disorders, cardiogenetics, and genetics of neurodevelopmental disorders.

**Marco Castori (MD, PhD)** is a clinical and molecular laboratory geneticist, Chief of the Division of Medical Genetics at Fondazione IRCCS-Casa Sollievo della Sofferenza (Italy). He is also the clinical coordinator of the Institutional Program on Rare Diseases and Member of the European Reference Networks for rare and complex diseases ERN-SKIN and ReCONNET. His main research interests comprise joint hypermobility, hereditary connective tissue disorders, hereditary bone and cardiovascular disorders, and developmental genetics.

## Data Availability

No new data were generated or analysed in support of this review.
